# VB-ROI-focused segmentation-to-morphometry workflow for vertebrobasilar dolichoectasia assessment on MRA

**DOI:** 10.3389/fneur.2026.1943260

**Published:** 2026-10-09

**Authors:** Jianheng Guan, Zhihua Liu, Hongkai Qiu, Chuanming Chen, Shujuan Yang, Jinfeng Cui, Zhibin Dai, Jin Zhang

**Affiliations:** 1 Department of Neurology, Boai Hospital of Zhongshan, Zhongshan, Guangdong, China; 2 Department of Neurosurgery, Zhongshan Torch Development Zone People's Hospital, Zhongshan, Guangdong, China

**Keywords:** magnetic resonance angiography, morphometric quantification, posterior circulation infarction, segmentation-to-morphometry, VB-ROI, vertebrobasilar dolichoectasia, vessel segmentation

## Abstract

**Introduction:**

Vertebrobasilar dolichoectasia (VBD) is closely associated with posterior circulation infarction; however, reliable MRA-based morphometric quantification is hindered by the challenges of delineating the basilar artery (BA) and bilateral vertebral arteries (R-VA and L-VA). These vessels are characterized by their thin, elongated structure, spatial localization, and high sensitivity to boundary deviations, endpoint instability, and centerline discontinuity, which render generic full-image vessel segmentation suboptimal for VBD-oriented morphometric analysis.

**Methods:**

In this study, we address this clinical-computational mismatch using a vertebrobasilar region-of-interest (VB-ROI)-focused segmentation-to-morphometry workflow. Initially, a full-image nnU-Net is employed for coarse anatomical localization, from which a prediction-derived VB-ROI is generated. Subsequently, a second nnU-Net performs fine segmentation of the BA/R-VA/L-VA within this anatomically focused field of view, and the restored full-size mask is utilized for segmentation evaluation and downstream morphometric quantification.

**Results:**

Compared to the direct full-image nnU-Net, the proposed two-stage VB-ROI workflow improved the mean Dice coefficient from 0.7974 to 0.9681, the mean Intersection over Union (IoU) from 0.7145 to 0.9412, and the mean centerline Dice from 0.7398 to 0.9206, while reducing the Hausdorff Distance at 95th percentile (HD95) from 8.6245 mm to 1.1709 mm. The prediction-derived VB-ROI demonstrated performance comparable to the ground-truth-based ROI setting without necessitating test-time ground truth, thus supporting the robustness of the localization strategy. Morphometric analysis indicated strong agreement in diameter-related measurements, while length, displacement, tortuosity, and curvature-related indices exhibited greater sensitivity to centerline extraction and endpoint definition.

**Discussion:**

These findings illustrate the technical feasibility of VB-ROI-focused segmentation for MRA-based vertebrobasilar morphometric quantification and provide a methodological foundation for future studies on VBD and posterior circulation infarction.

## 1 Introduction

Vertebrobasilar dolichoectasia (VBD) is characterized by abnormal dilation, elongation, displacement, and tortuosity of the vertebrobasilar arterial system. It has been associated with posterior circulation ischemic events, compressive symptoms, hemorrhagic complications, and adverse long-term outcomes (1–3). Due to the primarily morphological nature of these abnormalities, imaging-based assessment of VBD relies on the accurate delineation of the basilar artery (BA), right vertebral artery (R-VA), and left vertebral artery (L-VA). This is followed by reproducible measurements of vessel diameter, centerline length, lateral displacement, and tortuosity. Among the complications associated with VBD, posterior circulation infarction is of particular clinical concern, as variations in basilar artery caliber, elongation, and displacement have been reported to correlate with an increased ischemic risk in the vertebrobasilar territory (4–7). Consequently, reliable morphometric quantification of the vertebrobasilar system is a prerequisite for investigating the relationship between VBD morphology and posterior circulation infarction (6, 7).

Despite the clinical significance of these morphometric features, most automatic cerebrovascular segmentation methods are not specifically designed for quantifying VBD. While generic full-image vessel segmentation may achieve acceptable global overlap, VBD analysis relies on a limited set of anatomically specific vessels, namely the basilar artery (BA), right vertebral artery (R-VA), and left vertebral artery (L-VA). These vessels occupy only a small portion of the MRA volume and possess thin, elongated, and curved structures. Consequently, segmentation errors that may appear minor at the voxel level can considerably impact centerline extraction, local diameter estimation, endpoint definition, lateral displacement measurement, and tortuosity calculation. The methodological challenge thus lies not merely in segmentation accuracy, but in determining whether the predicted masks are sufficiently stable to support downstream VBD-oriented morphometric quantification.

Automatic cerebrovascular segmentation from MRA presents an effective strategy foralleviating the manual measurement burden and enhancing reproducibility. Self-configuring segmentation frameworks, such as nnU-Net, have emerged as robust general-purpose baselines for biomedical image segmentation (8). Furthermore, vessel-specific networks and topology-aware metrics have significantly advanced the segmentation and evaluation of tubular anatomical structures (9, 10). However, direct full-image segmentation remains inadequately aligned with the requirements of BA/R-VA/L-VA morphometric analysis. Within the complete MRA space, the model must differentiate small target vessels from extensive irrelevant backgrounds, adjacent non-target vasculature, and weak-signal vessel segments. This clinical-computational mismatch can result in fragmented vertebral artery predictions, incomplete distal coverage, endpoint instability, and boundary outliers. Such inaccuracies are particularly detrimental to VBD-oriented morphometry, as downstream measurements rely not only on voxel overlap but also on centerline continuity, endpoint stability, and local caliber accuracy.

In this study, we address this clinical-computational mismatch using a vertebrobasilar region-of-interest (VB-ROI)-focused segmentation-to-morphometry workflow. The proposed workflow separates VBD-oriented MRA analysis into two computational steps: coarse vertebrobasilar localization in the full image space and fine BA/R-VA/L-VA segmentation within an anatomically focused ROI. The full-image nnU-Net is used to generate a localization prior for defining the VB-ROI, whereas the second nnU-Net performs high-fidelity vessel delineation within the focused field of view. The restored second-stage mask is then used for segmentation evaluation and downstream morphometric quantification. This design is intended not to introduce a new network architecture, but to better align the segmentation setting with the anatomical and measurement requirements of VBD assessment. By restricting fine segmentation to the VB-ROI, the target vessels occupy a larger fraction of the input space, reducing irrelevant anatomical interference and improving the stability of vessel boundaries, centerlines, and endpoints. Therefore, this study aimed to develop and validate a VB-ROI-focused segmentation-to-morphometry framework for automated and reliable quantitative assessment of vertebrobasilar dolichoectasia.

## 2 Related work

### 2.1 VBD-related imaging criteria and morphometric assessment

VBD is commonly characterized by abnormal dilatation, elongation, displacement, and tortuosity of the vertebrobasilar arterial system (3, 11). Because these abnormalities are primarily morphological, imaging-based assessments have traditionally relied on interpretable geometric measurements, including the basilar artery (BA) diameter, BA length, lateral displacement of the BA, vertebral artery (VA) length, and VA deviation (12). Classical diagnostic criteria primarily define BA ectasia based on vessel caliber, while dolichosis-related criteria further incorporate vessel elongation and lateral displacement (1, 13). These measurements provide clinically meaningful descriptors of vertebrobasilar morphology; however, they are typically obtained manually or semi-manually, making them heavily reliant on accurate delineation of the BA and bilateral VAs. This dependency establishes a direct methodological link between vessel segmentation and VBD-oriented morphometric quantification. Diameter-related indices are primarily influenced by local boundary accuracy, as small deviations in boundary delineation can significantly alter the estimated maximum or mean vessel caliber. In contrast, indices related to length, displacement, tortuosity, and curvature impose stricter requirements on the predicted mask, necessitating vessel continuity, centerline stability, and the preservation of anatomically meaningful endpoints. Therefore, VBD-oriented segmentation should not be viewed solely as a voxel-overlap problem; it must also be assessed based on whether the resulting masks can support reliable downstream morphometric measurements.

### 2.2 Automated cerebrovascular segmentation from MRA

Deep learning has significantly advanced the field of automatic cerebrovascular segmentation from Magnetic Resonance Angiography (MRA) (14). General-purpose self-configuring frameworks, such as nnU-Net, have established robust baselines for biomedical image segmentation by automatically adapting preprocessing, network configuration, training, and post-processing to specific tasks (8). Vessel-specific methodologies, including BRAVE-NET, have further demonstrated the feasibility of fully automated segmentation of arterial brain vessels in patients with cerebrovascular diseases (9, 15). Furthermore, recent benchmarks in vascular segmentation and topology-aware evaluation strategies have underscored the importance of anatomical vessel labeling and topological preservation in cerebrovascular image analysis (16). Metrics such as clDice have been proposed to assess and enhance centerline-aware topology preservation in tubular structures (10). More recently, foundational segmentation models such as MedSAM have expanded the scope of general medical image segmentation; however, they do not directly address the vessel-specific topology, endpoint stability, and morphometric reliability necessary for quantification oriented toward VBD, BA/R-VA, and L-VA (17).

Despite these advances, most existing cerebrovascular segmentation studies are not fully aligned with VBD-oriented analysis. Their primary objectives typically focus on whole-brain vessel extraction, general arterial segmentation, or the anatomical segmentation of vascular networks, such as the Circle of Willis. Consequently, their evaluation often emphasizes global segmentation accuracy or the preservation of network-level topology. However, VBD-oriented morphometry relies on the stable delineation of a small set of target vessels, specifically the BA, R-VA, and L-VA. These vessels comprise only a small fraction of the MRA volume and are characterized by their thin, elongated, and spatially localized nature, making them susceptible to weak-signal interruptions. As a result, a model that performs reasonably well for whole-volume vessel segmentation may still produce fragmented, endpoint-unstable, or boundary-inaccurate masks for the BA, R-VA, and L-VA, which are unsuitable for downstream VBD morphometric quantification.

### 2.3 Gap between vessel segmentation and VBD-oriented quantification

The aforementioned studies highlight a clinical-computational gap between generic vessel segmentation and VBD-oriented morphometric analysis. Clinically, VBD assessment necessitates reproducible measurements of vertebrobasilar caliber, elongation, displacement, tortuosity, and vessel course. Computationally, most automatic segmentation methods are framed as full-image vessel extraction or anatomical labeling problems, rather than as morphometry-oriented segmentation tasks focused on the basilar artery (BA) and bilateral vertebral arteries (VAs). This discrepancy is significant, as morphometric errors can arise not only from imperfect network predictions but also from an ill-conditioned task setting where small target vessels are segmented within a large volume dominated by irrelevant background, adjacent non-target vasculature, and weak vessel signals. This gap cannot be fully resolved by merely optimizing global segmentation overlap or augmenting model capacity. For VBD-oriented analysis, the critical question is whether the segmentation task is spatially and anatomically aligned with the downstream measurements. Full-image segmentation requires the model to simultaneously address target localization, vessel discrimination, and fine boundary delineation within a large image space. In contrast, VBD morphometry demands stable fine delineation of BA/R-VA/L-VA structures after the vertebrobasilar field of view has been reliably localized. This distinction necessitates a localization-to-segmentation task decomposition rather than a straightforward cascade of segmentation models.

In this study, we address the existing gap by reformulating VBD-oriented MRA analysis as a segmentation-to-morphometry problem focused on VB-ROI. A full-image nnU-Net is utilized solely to generate a coarse anatomical localization prior and to define a prediction-derived VB-ROI; its output is not employed as the final vessel mask or for morphometric measurement. Subsequently, a second nnU-Net performs fine BA/R-VA/L-VA segmentation within this anatomically focused field of view. This design specifically targets the requirements of VBD-oriented analysis, including stable thin-vessel delineation, centerline preservation, boundary accuracy, endpoint stability, and reliable downstream morphometric quantification.

## 3 Methods

### 3.1 Problem formulation

The goal of this study is not generic whole-brain vessel segmentation, but a clinically oriented segmentation-to-quantification workflow for VBD-oriented analysis. VBD assessment mainly depends on the morphology of the vertebrobasilar arterial system, particularly BA, R-VA, and L-VA. Therefore, we reformulate the original multi-label cerebral vessel segmentation problem into a VBD-specific anatomical segmentation task focusing on these three target vessels.

Given an input MRA volume I, the objective is to estimate a label map S:

$$\begin{array}{l} \text{S}\left(\text{Ω}\right)in\left\{0,1,23,24\right\}, \end{array}$$

where 0 denotes background, 1 denotes BA, 23 denotes R-VA, and 24 denotes L-VA. As formalized in Equation 1, the predicted segmentation is then used to derive VBD-oriented morphometric quantities:

$$\begin{array}{l} \text{M}\left(\text{S}\right)=\left\{\text{dmax},\text{dmean},\text{lcl},\text{slat},\text{tau},\text{kappa}\right\}, \end{array}$$

where dmax is maximum vessel diameter, dmean is mean centerline diameter, lcl is centerline length, slat is lateral shift, tau is tortuosity, and kappa denotes curvature-related measurements. This formulation explicitly couples segmentation accuracy with downstream morphometric quantification in Equation 2, so that geometric measurement error can be attributed to and analyzed against segmentation quality.

### 3.2 Dataset and label definition

We used TopBrain-MRA data formatted for nnU-Net. The original TopBrain annotation contains multiple cerebral vessel labels, but this study focuses on the three anatomical structures most relevant to VBD-oriented analysis: BA, R-VA, and L-VA. Their original TopBrain label IDs are BA = 1, R-VA = 23, and L-VA = 24. Experiments were conducted using a fixed train/validation split, and all reported quantitative results were computed on the held-out validation split.

The dataset and label definitions are summarized in Table 1.

**Table 1:** Dataset and label setting.

Dataset	Modality	Split	Labels used
TopBrain–MRA	MRA	Fixed train/validation split	BA=1, R–VA=23, L–VA=24

The evaluated segmentation strategies were compared to clarify the contribution of the proposed two-stage VB-ROI workflow relative to conventional full-image segmentation. The comparison settings are summarized in Table 2.

**Table 2:** Comparison of evaluated segmentation workflows.

Setting	ROI source	Test–time GT used?	Role
Full–image nnU–Net	None	No	Baseline
GT–based VB–ROI	Ground–truth mask	Yes	Oracle upper bound
Two–stage VB–ROI	Stage−1 prediction	No	Practical workflow

For ROI-focused training, the original labels were remapped to a compact four-class representation:

SROI(x)ε{0,1,2,3},0=background, 1=BA, 2=R-VA, 3=L-VA, (3)

This compact representation defined in Equation 3 avoids unnecessary output channels during ROI training and forces the model to concentrate on VBD-relevant vessels. After ROI inference, predictions were restored to the original image space and remapped back to the original TopBrain label IDs according to Equation 4:

$$\begin{array}{l} 1->1,2->23,3->24, \end{array}$$

### 3.3 VB-ROI focused workflow

The proposed workflow is motivated by the anatomical characteristics of VBD-related vessels. Unlike large organs or compact lesions, BA and bilateral VA are thin tubular structures with small volume, long centerline extent, and high sensitivity to local discontinuity. In full-image segmentation, the model must solve a highly imbalanced problem dominated by irrelevant background and non-target vessels. This setting may produce fragmented VA predictions or small boundary outliers, both of which can substantially affect downstream measurements such as centerline length, lateral shift, tortuosity, and diameter estimation.

Therefore, the core design of this study is not to introduce a new segmentation network, but to reformulate the spatial context of the segmentation problem. The full-image model is used to provide coarse anatomical localization, whereas the second-stage ROI model performs fine segmentation within a constrained vertebrobasilar field of view. This design directly targets the VBD-oriented analysis bottleneck: stable delineation of BA, R-VA, and L-VA for subsequent morphometric quantification.

Figure 1 illustrates the complete workflow. The full-image nnU-Net first provides either a direct baseline prediction or a coarse vertebrobasilar mask for ROI localization. A prediction-derived VB-ROI is then generated by computing the bounding box of the predicted BA/R-VA/L-VA region and expanding it with a predefined physical margin. The ROI nnU-Net performs fine segmentation within this focused field of view. Finally, the ROI prediction is restored to the original image space and used for segmentation evaluation, morphometric measurement, and threshold-based consistency analysis.

**Figure 1:**
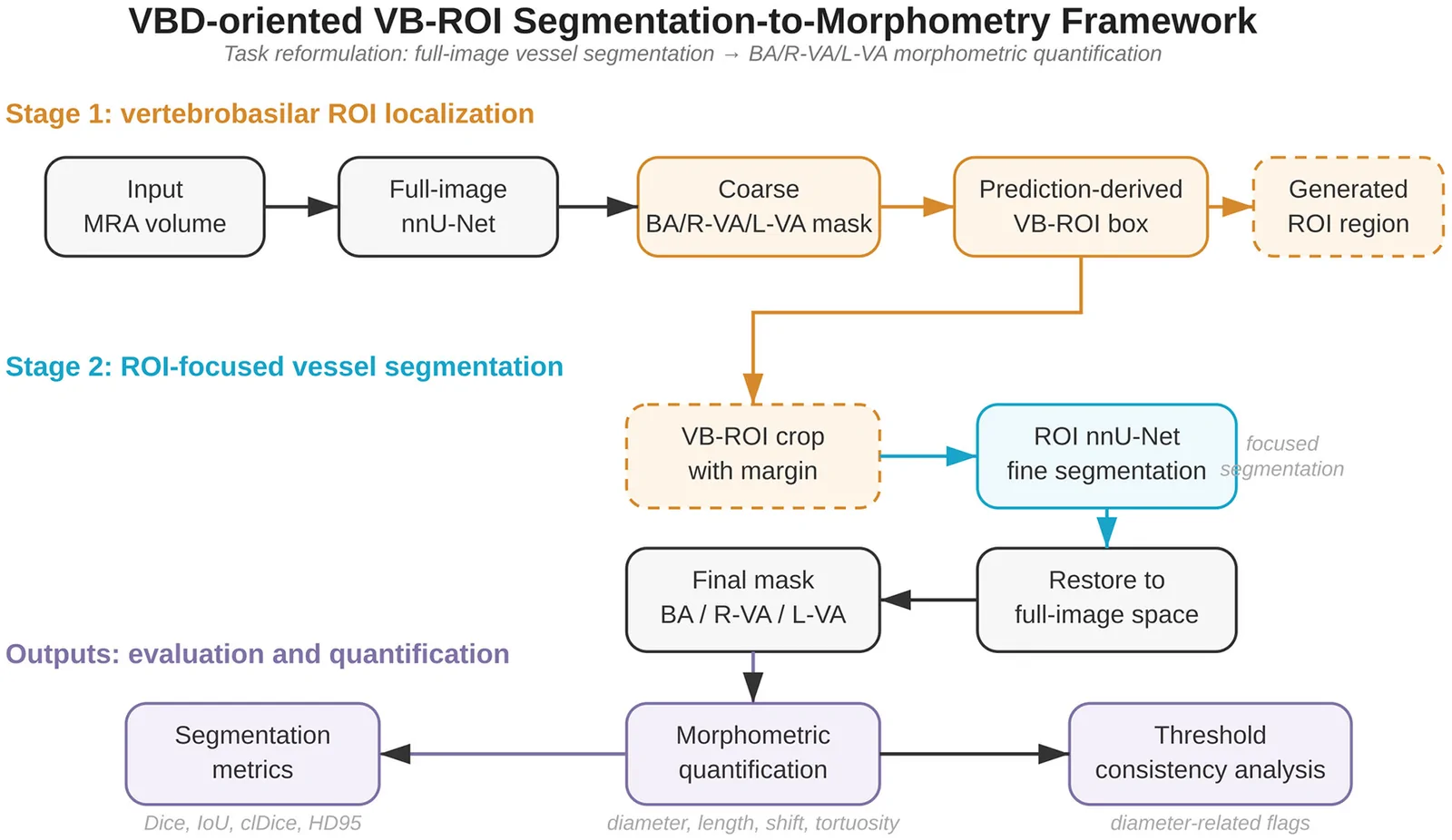
Overview of the proposed VBD-oriented VB-ROI focused segmentation-to-morphometry workflow. The method does not introduce a new network architecture; instead, it reformulates VBD-oriented analysis from generic full-image vessel segmentation into clinically targeted BA/R-VA/L-VA segmentation and morphometric quantification. The full-image nnU-Net provides coarse anatomical localization, the prediction-derived VB-ROI constrains the fine segmentation stage, and the restored full-size mask is used for segmentation evaluation, morphometric analysis, and threshold-based consistency analysis.

### 3.4 Evaluated segmentation settings

We evaluated three settings. First, a full-image nnU-Net was trained and evaluated in the original image space. This setting serves as the baseline and measures the performance of direct full-volume segmentation. Second, a GT-based VB-ROI setting was constructed by generating the ROI bounding box from the ground-truth BA/R-VA/L-VA mask. This oracle setting is not intended for deployment; it isolates the effect of focusing on the vertebrobasilar region. Third, a prediction-derived two-stage VB-ROI workflow was used as the practical inference pipeline. In this setting, the ROI is generated from a coarse full-image prediction rather than from ground truth.

### 3.5 Segmentation evaluation

Because VBD-related vessels are thin and elongated, segmentation evaluation should not rely only on volumetric overlap. We therefore evaluated each target vessel using overlap, centerline, boundary, detection, and connectedness-aware metrics. Dice and intersection-over-union (IoU) measure volumetric overlap. Centerline Dice (clDice) measures whether the predicted vessel preserves tubular centerline structure (10). HD95 and ASD were computed in millimeters using image spacing. Precision and recall characterize false-positive and false-negative behavior. Connected-component error in Equation 5 was defined as:

$$\begin{array}{l} Ecc=|Npred-Ngt|, \end{array}$$

where Npred and Ngt denote the number of connected components in the predicted and ground-truth masks.

### 3.6 Morphometric quantification

For each predicted and ground-truth vessel mask, we extracted a centerline and computed VBD-oriented morphometric measurements. Maximum diameter was estimated from the local vessel radius derived from the binary mask. Mean centerline diameter was computed by averaging diameter estimates along centerline points. Centerline length was computed as the accumulated Euclidean distance along the extracted centerline. Lateral shift was estimated as the maximum deviation of the centerline from the straight chord connecting its endpoints. Tortuosity was defined as the ratio between centerline length and chord length. Curvature-related measurements were computed from local changes in centerline direction.

Prediction-derived measurements were compared with ground-truth-derived measurements using mean absolute error (MAE), Pearson correlation coefficient, and intraclass correlation coefficient (ICC). Importantly, centerline length, lateral shift, tortuosity, and curvature were computed from the available annotated BA/R-VA/L-VA segments. They should therefore be interpreted as segmentation-derived geometric descriptors rather than complete clinical measurements of the entire vertebrobasilar course. Diameter-related measurements are emphasized because they depend less on global vessel extent and on endpoint definition, and therefore constitute the most transferable morphometric descriptors within the segment-level annotation regime of this study.

### 3.7 Threshold-based consistency analysis

We further performed a threshold-based consistency analysis between prediction-derived and ground-truth-derived morphometric flags. The evaluated criteria included BA maximum diameter greater than 4.5 mm and several length- or displacement-related thresholds reported in VBD literature (1, 3, 11, 13). However, because the current annotations cover target vessel segments and the validation split contains limited positive cases for several endpoints, this analysis is interpreted as preliminary consistency assessment rather than definitive clinical validation. Endpoints without positive reference cases are reported as not applicable for sensitivity or kappa and should be viewed mainly as false-positive control.

### 3.8 Training configuration

The proposed framework consisted of two nnU-Net-based stages. For the VB-ROI localization stage (Dataset701_TopBrainMR), nnU-Net v2 was implemented using a 3D full-resolution configuration with nnUNetTrainer and nnUNetPlans. The available records confirmed training of fold 0 using the commands “nnUNetv2_plan_and_preprocess -d 701 -c 3d_fullres –verify_dataset_integrity” and “nnUNetv2_train 701 3d_fullres 0”. After searching the retained project files, historical records, experiment outputs, and tracking information, the original Stage-1 plans, checkpoints, debug files, training logs, and tracking records containing low-level optimization parameters were not available. Therefore, patch size, batch size, training epochs, initial learning rate, optimizer, learning-rate scheduler, loss function, and augmentation strategy could not be reliably recovered and were not retrospectively inferred.

For the targeted vertebrobasilar vessel segmentation stage (Dataset801_VBROI_GT), nnU-Net v2 was configured with a 3D full-resolution PlainConvUNet architecture (nnUNetTrainer, nnUNetPlans). The patch size was 80 × 128 × 192 voxels, with a batch size of 2. The model was trained for 1,000 epochs using an initial learning rate of 0.01 with SGD optimization (momentum 0.99, Nesterov acceleration, and weight decay of 3 × 10^−5^). The learning rate was adjusted using PolyLRScheduler, and the loss function was deep-supervised DC_and_CE_loss consisting of MemoryEfficientSoftDiceLoss and RobustCrossEntropyLoss.

## 4 Experimental results

### 4.1 Segmentation performance

Figure 2 summarizes the main segmentation results. The full-image nnU-Net achieved strong BA segmentation but showed substantially lower performance for R-VA and L-VA, reflecting the difficulty of segmenting thin vertebral arteries in the full image space. In contrast, ROI-focused methods markedly improved both overlap and centerline-aware metrics. The prediction-derived two-stage VB-ROI workflow achieved performance comparable to the GT-based ROI setting, suggesting that coarse full-image prediction is sufficient for practical ROI localization and that the primary performance gain comes from focused vertebrobasilar segmentation.

**Figure 2:**
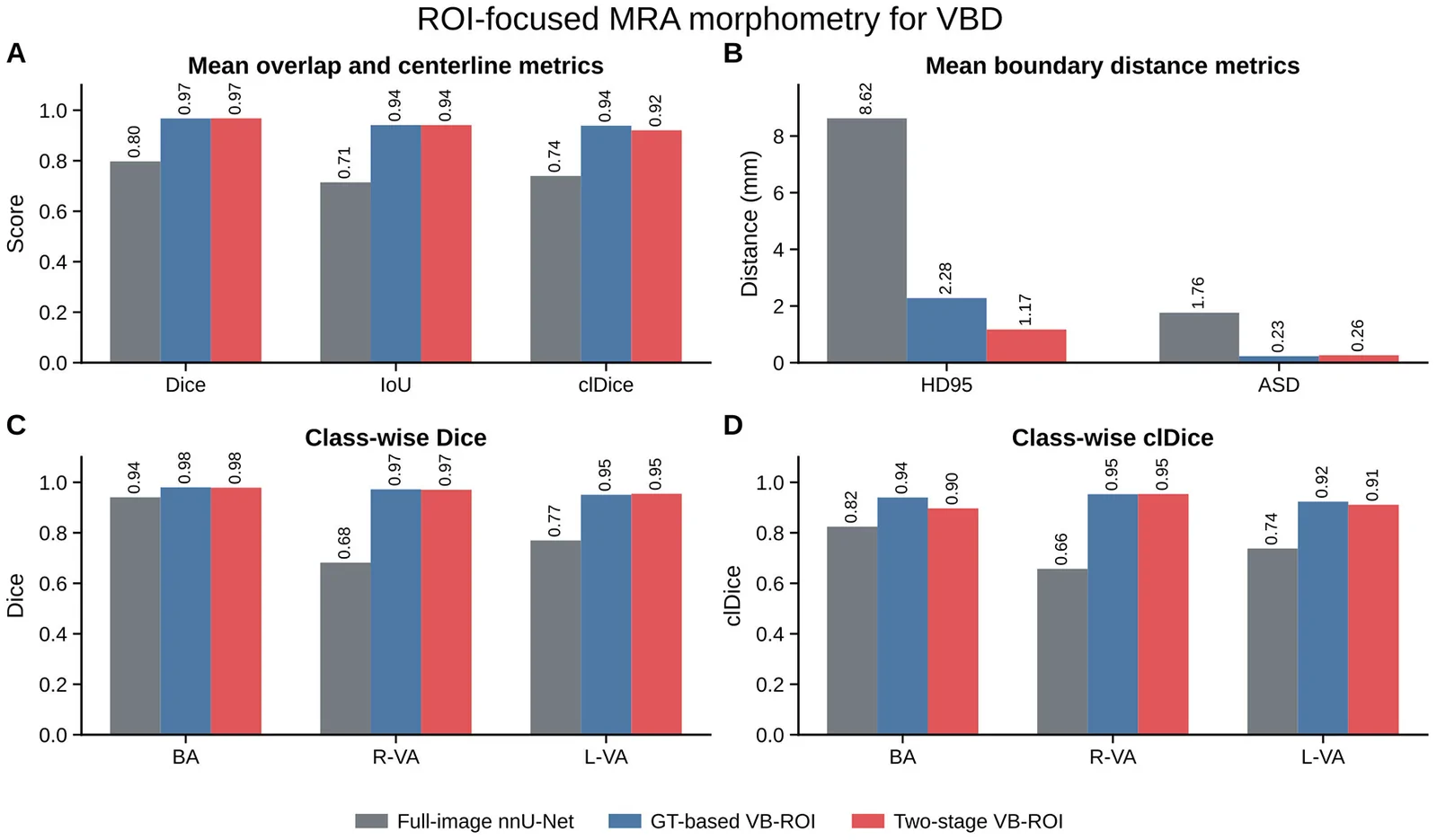
Segmentation performance overview for VBD-relevant vessels. Compared with full-image nnU-Net, both GT-based VB-ROI and prediction-derived two-stage VB-ROI increase mean Dice, IoU, and clDice while reducing HD95 and ASD. The improvement is especially pronounced for R-VA and L-VA, indicating that ROI-focused segmentation directly addresses the instability of full-image segmentation for thin vertebral arteries. GT-based VB-ROI denotes an oracle upper-bound setting, whereas the two-stage VB-ROI setting is the practical inference workflow.

Compared with full-image nnU-Net, the two-stage VB-ROI workflow improved mean Dice from 0.7974 to 0.9681, mean IoU from 0.7145 to 0.9412, and mean clDice from 0.7398 to 0.9206. Boundary errors were also substantially reduced, with mean HD95 decreasing from 8.6245 mm to 1.1709 mm and mean ASD decreasing from 1.7635 mm to 0.2627 mm. The largest gains were observed for the vertebral arteries. R-VA Dice increased from 0.6818 to 0.9707, and L-VA Dice increased from 0.7697 to 0.9547. These results support the central hypothesis that focusing the segmentation field of view on the vertebrobasilar region is more effective than directly segmenting small target vessels in the full image space.

Detailed class-wise segmentation metrics are provided in Table 3.

**Table 3:** Detailed segmentation performance of BA, R–VA, and L–VA.

Method	Class	Dice	IoU	clDice	HD95	ASD	Precision	Recall	Comp. Err.
Full– image nnU– Net	BA	0.9407 ± 0.0230	0.8888 ± 0.0407	0.8244 ± 0.1473	0.6398 ± 0.3008	0.1249 ± 0.0504	0.9492 ± 0.0462	0.9352 ± 0.0441	1.0000 ± 1.8257
Full– image nnU–Net	R–VA	0.6818 ± 0.3263	0.5875 ± 0.3352	0.6572 ± 0.3138	6.2964 ± 6.6239	1.9613 ± 2.6167	0.8358 ± 0.2889	0.6227 ± 0.3625	1.8571 ± 1.6762
Full–image nnU–Net	L–VA	0.7697 ± 0.2276	0.6673 ± 0.2658	0.7377 ± 0.1540	18.9374 ± 16.9979	3.2042 ± 2.9375	0.7531 ± 0.2860	0.8305 ± 0.1533	2.7143 ± 1.9760
Full–image nnU–Net	Mean	0.7974 ± 0.1499	0.7145 ± 0.1693	0.7398 ± 0.1287	8.6245 ± 7.4310	1.7635 ± 1.7079	0.8460 ± 0.1210	0.7961 ± 0.1460	1.8571 ± 1.0157
GT–based VB–ROI	BA	0.9800 ± 0.0094	0.9609 ± 0.0179	0.9399 ± 0.0333	0.3393 ± 0.1122	0.0402 ± 0.0241	0.9772 ± 0.0062	0.9829 ± 0.0059	0.1429 ± 0.3780
GT–based VB–ROI	R–VA	0.9721 ± 0.0390	0.9481 ± 0.0693	0.9529 ± 0.0425	4.8223 ± 12.3667	0.3570 ± 0.8951	0.9715 ± 0.0401	0.9728 ± 0.0381	0.7143 ± 1.4960
GT–based VB–ROI	L–VA	0.9506 ± 0.0839	0.9150 ± 0.1339	0.9235 ± 0.1392	1.6746 ± 3.9095	0.2914 ± 0.7105	0.9707 ± 0.0312	0.9362 ± 0.1235	0.5714 ± 1.1339
GT–based VB–ROI	Mean	0.9676 ± 0.0436	0.9413 ± 0.0727	0.9388 ± 0.0558	2.2787 ± 5.4624	0.2295 ± 0.5424	0.9731 ± 0.0286	0.9640 ± 0.0537	0.4762 ± 0.8357
Two– stage VB– ROI	BA	0.9788 ± 0.0100	0.9586 ± 0.0189	0.8968 ± 0.0770	0.3402 ± 0.1146	0.1692 ± 0.2656	0.9761 ± 0.0170	0.9816 ± 0.0052	0.7143 ± 0.7559
Two–stage VB–ROI	R–VA	0.9707 ± 0.0370	0.9450 ± 0.0656	0.9538 ± 0.0419	0.5125 ± 0.8439	0.2651 ± 0.6442	0.9770 ± 0.0223	0.9649 ± 0.0510	0.5714 ± 1.1339
Two–stage VB–ROI	L–VA	0.9547 ± 0.0703	0.9200 ± 0.1155	0.9112 ± 0.1685	2.6601 ± 6.5159	0.3538 ± 0.8710	0.9641 ± 0.0480	0.9466 ± 0.0906	1.0000 ± 2.6458
Two–stage VB–ROI	Mean	0.9681 ± 0.0386	0.9412 ± 0.0658	0.9206 ± 0.0800	1.1709 ± 2.4888	0.2627 ± 0.5905	0.9724 ± 0.0284	0.9643 ± 0.0476	0.7619 ± 1.3012

Detailed segmentation performance for BA, R–VA, L–VA, and their mean. HD95 and ASD are reported in millimeters.

### 4.2 Qualitative segmentation visualization

Qualitative results in Figure 3 further support the quantitative findings. Full-image nnU-Net can delineate BA reasonably well, but VA predictions are more prone to discontinuity or incomplete coverage. The VB-ROI workflow reduces irrelevant anatomical interference and yields more coherent BA/R-VA/L-VA masks, which is important for downstream centerline extraction and morphometric measurement.

**Figure 3:**
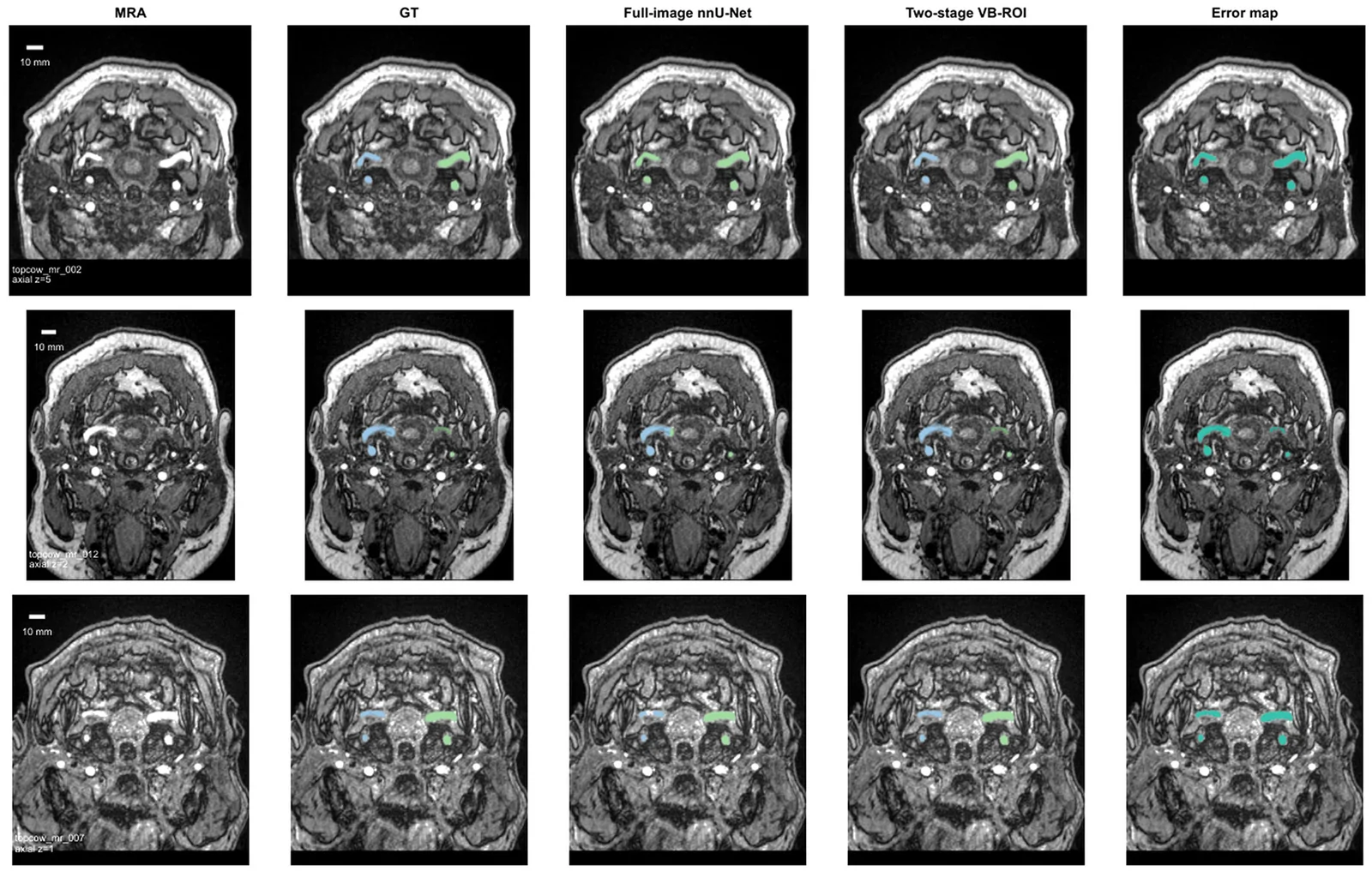
Representative 2D overlay visualization. Each row shows the original MRA slice, ground truth, full-image nnU-Net prediction, two-stage VB-ROI prediction, and error map. BA, R-VA, and L-VA are shown in red, blue, and green, respectively. The error map highlights true positives, false positives, and false negatives. Compared with full-image segmentation, the two-stage VB-ROI workflow produces more stable vertebrobasilar vessel delineation with fewer discontinuities and boundary outliers. Source: TopBrain Challenge Data Release, Zenodo, doi: 10.5281/zenodo.16878417.

The 3D surface rendering in Figure 4 illustrates the anatomical effect of ROI focusing. Because VBD-oriented assessment depends on the global course and local caliber of the vertebrobasilar arteries, preserving three-dimensional vessel continuity is more informative than improving voxel overlap alone. The two-stage VB-ROI prediction shows a closer geometric match to the reference vessel surface, supporting its use for morphometric analysis.

**Figure 4:**
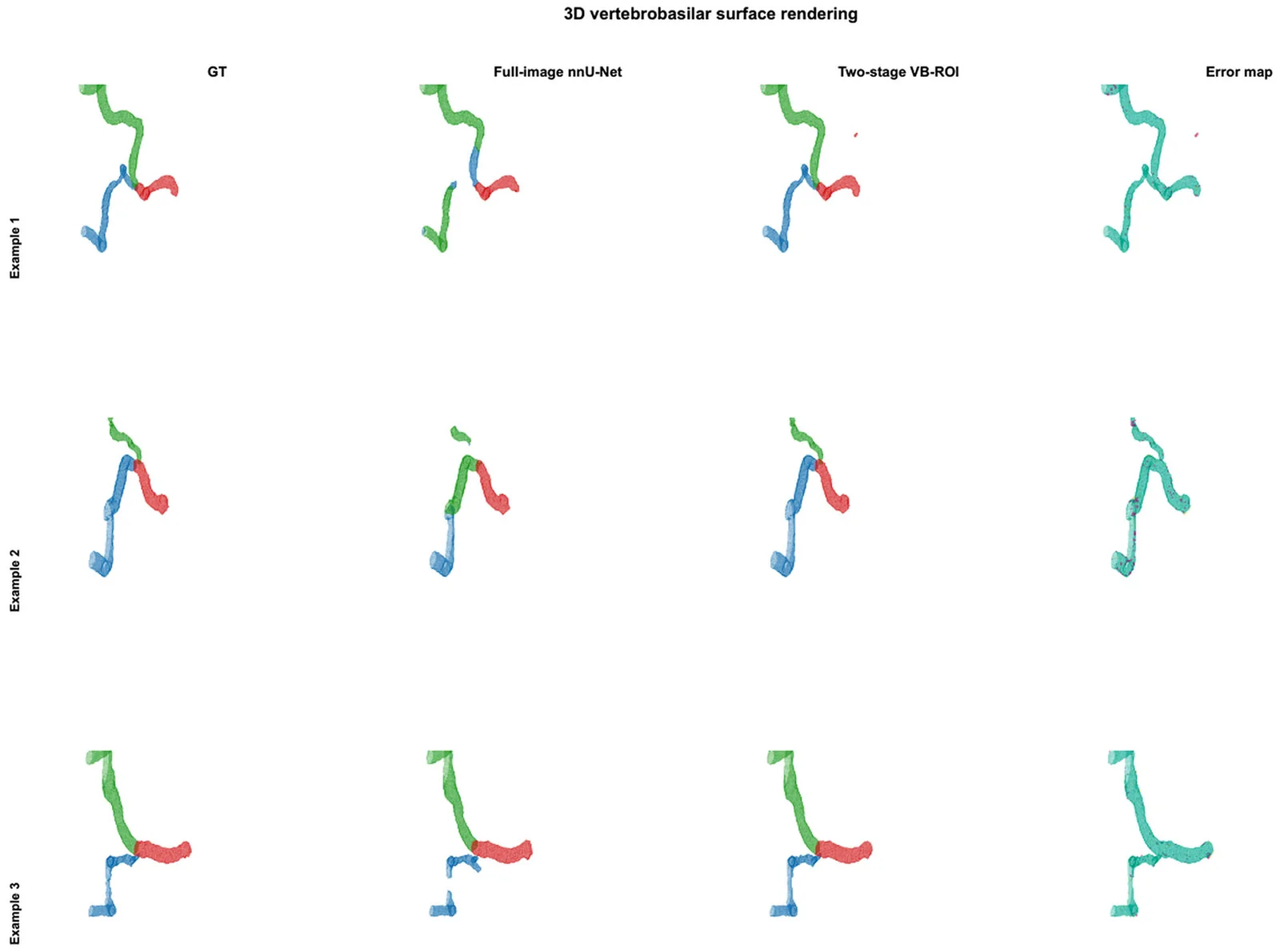
Three-dimensional surface rendering of BA, R-VA, and L-VA. The GT mask, full-image nnU-Net prediction, two-stage VB-ROI prediction, and error map are shown from the same viewpoint. The 3D visualization highlights the tubular continuity of the vertebrobasilar system and demonstrates that ROI-focused segmentation better preserves vessel geometry, which is critical for centerline and diameter measurements. Source: TopBrain Challenge Data Release, Zenodo, doi: 10.5281/zenodo.16878417.

### 4.3 ROI localization analysis

Figure 5 visualizes the role of the first stage in the proposed workflow. The coarse full-image prediction is not intended to be the final segmentation result; instead, it provides a practical anatomical prior for localizing the vertebrobasilar ROI. This explains why the prediction-derived two-stage setting can approach the GT-based ROI upper bound: the first stage only needs to localize the relevant region sufficiently well, while the second-stage ROI model performs the high-fidelity vessel segmentation. The robustness of this localization step to the ROI margin is quantified in Section 4.4 (Table 4, Figure 6).

**Figure 5:**
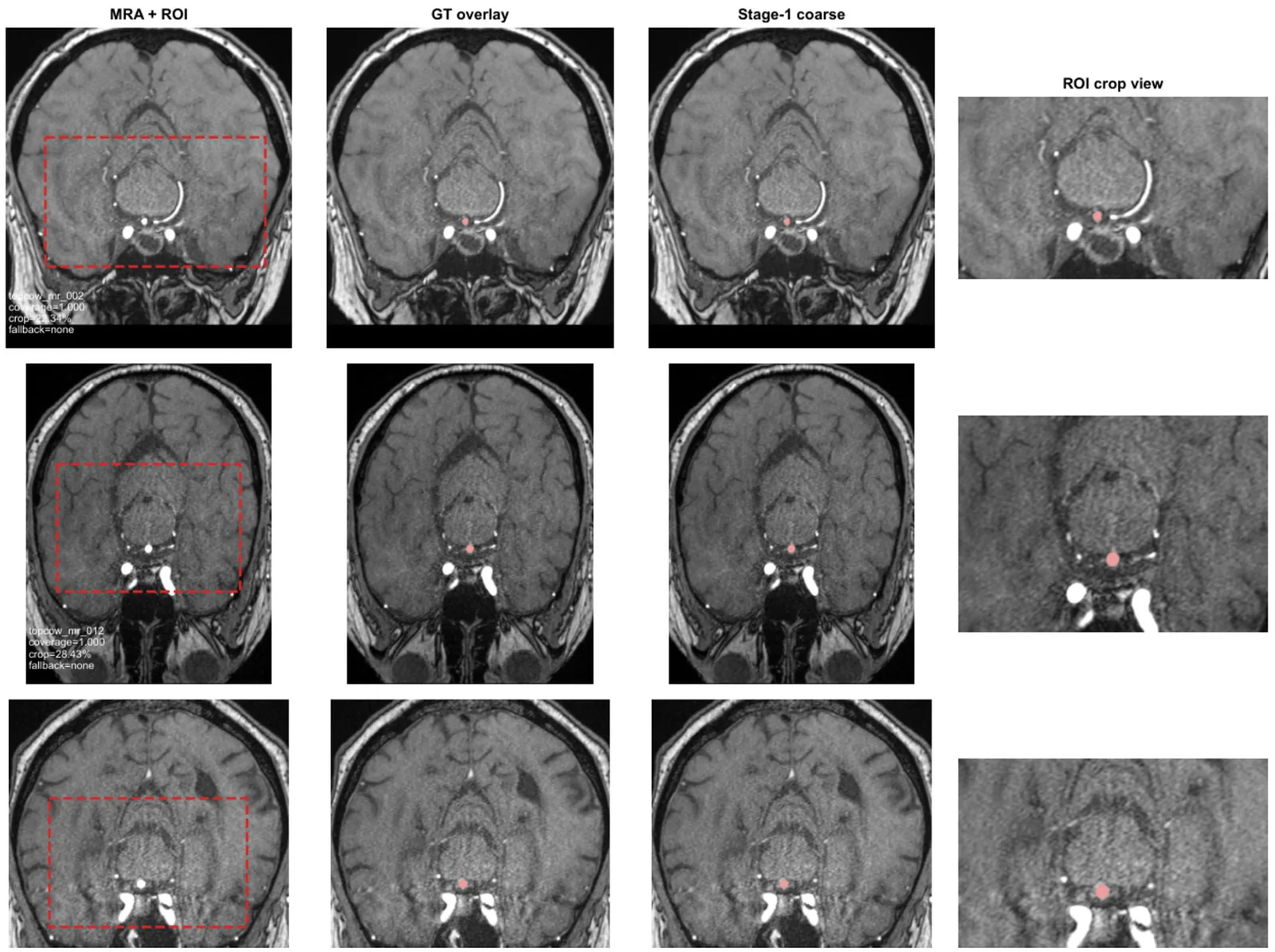
Prediction-derived VB-ROI localization analysis. The figure shows the original MRA, GT overlay, Stage-1 coarse prediction, prediction-derived ROI bounding box, and cropped ROI view. Stage 1 is used for anatomical localization rather than final segmentation. The resulting ROI provides a focused field of view for Stage-2 fine segmentation while preserving the BA/R-VA/L-VA region required for VBD-oriented analysis. Source: TopBrain Challenge Data Release, Zenodo, doi: 10.5281/zenodo.16878417.

**Table 4:** Sensitivity analysis of prediction–derived VB–ROI margins.

Margin (mm)	Mean GT coverage	Min GT coverage	Crop ratio	Pred–GT ROI IoU
0	0.9976	0.9834	0.0573	0.9814
5	1.0000	1.0000	0.0818	0.9835
10	1.0000	1.0000	0.1293	0.9868
15	1.0000	1.0000	0.1949	0.9885
20	1.0000	1.0000	0.2765	0.9897
25	1.0000	1.0000	0.3658	0.9929
30	1.0000	1.0000	0.4565	0.9936

GT coverage denotes the fraction of ground-truth BA/R-VA/L-VA voxels enclosed by the prediction-derived ROI. Crop ratio denotes the ROI volume divided by the full-image volume. Pred-GT ROI IoU measures the overlap between the prediction-derived ROI and the GT-derived ROI under the same margin.

**Figure 6:**
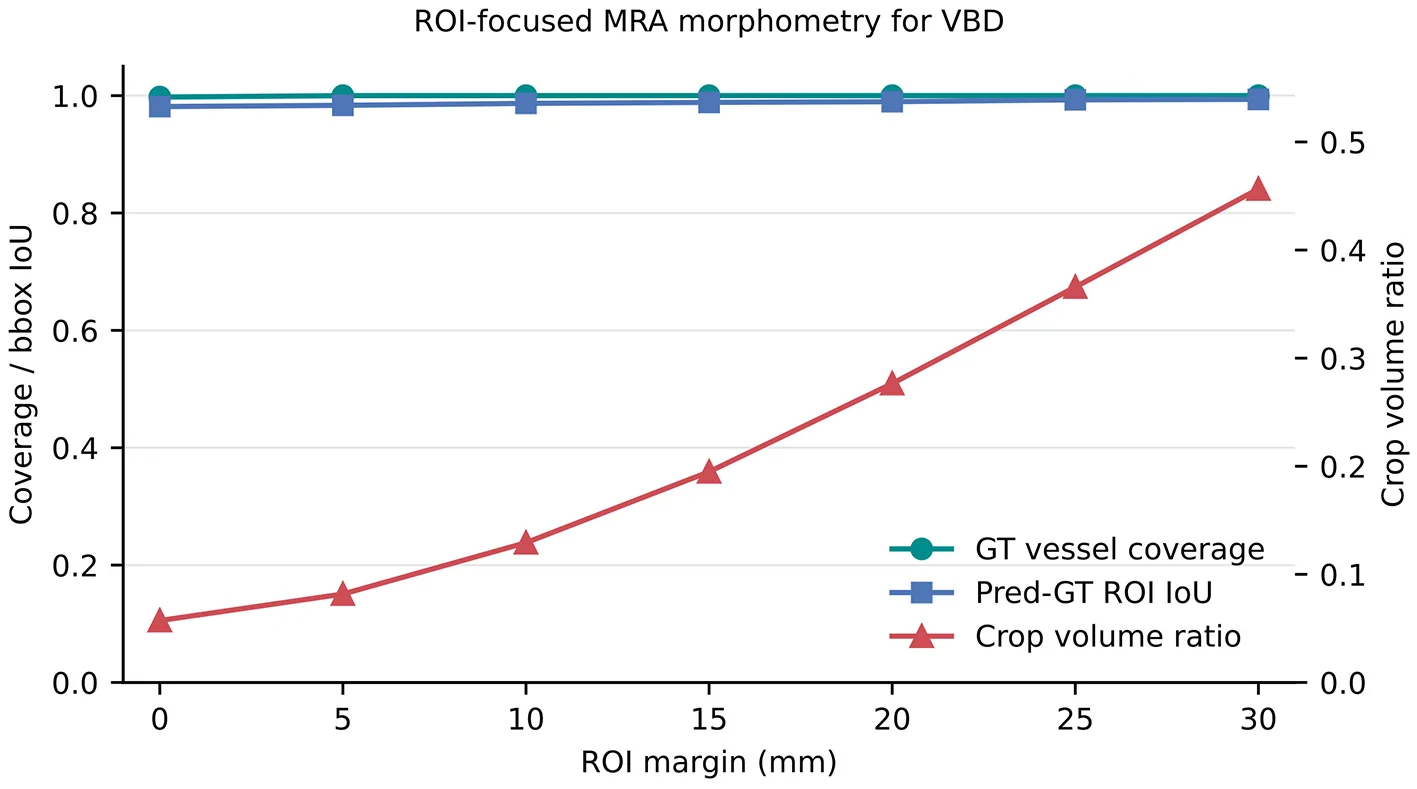
ROI margin sensitivity analysis. A small physical margin already allows the prediction-derived VB-ROI to fully enclose the target vertebrobasilar vessels, whereas larger margins progressively enlarge the crop volume without improving coverage.

### 4.4 ROI margin sensitivity analysis

To assess whether the prediction-derived VB-ROI is robust to the choice of physical margin, we conducted a margin sensitivity analysis. The Stage-1 full-image nnU-Net prediction was held fixed and used solely for ROI localization. We then generated VB-ROI boxes under a range of physical margins and measured whether each resulting ROI fully enclosed the ground-truth BA, R-VA, and L-VA masks. This analysis isolates the localization safety and crop efficiency of the ROI-generation step; it does not retrain or re-execute the second-stage segmentation model for each margin, and therefore characterizes the geometric robustness of localization rather than end-to-end segmentation accuracy.

Without any physical margin, the prediction-derived ROI omitted a small fraction of ground-truth vessel voxels, with a minimum GT coverage of 0.9834. This indicates that a tight Stage-1 bounding box is vulnerable to small boundary errors and endpoint under-segmentation in the coarse prediction, most notably for thin vertebral-artery segments. A 5 mm margin was sufficient to achieve complete GT vessel coverage across the validation split, at a mean crop-volume ratio of only 0.0818. Enlarging the margin beyond this point did not improve coverage, which had already saturated, but it monotonically increased the cropped volume.

The main experiments used a 15 mm margin. This choice is deliberately conservative: it preserves complete BA/R-VA/L-VA coverage while supplying additional anatomical context to the second-stage ROI nnU-Net. Relative to 5 mm, the 15 mm margin raises the crop-volume ratio from 0.0818 to 0.1949, yet remains far below full-image inference and reduces the risk of truncating distal vessel endpoints. Overall, these results support the robustness of the prediction-derived ROI localization step and justify the selected margin as a safety-oriented compromise between anatomical coverage and computational focus.

### 4.5 VBD-oriented morphometric measurements

Beyond segmentation accuracy, Figure 7 quantifies the agreement between prediction-derived and ground-truth-derived continuous VBD-oriented morphometric measurements. The prediction-derived BA maximum diameter closely matches the ground-truth-derived value, which is important because BA diameter is a commonly used VBD-oriented imaging criterion. Length- and shift-related measurements show larger deviations, suggesting that these indices are more sensitive to centerline extraction, endpoint definition, and local segmentation variation. Therefore, diameter-related morphometry represents the most reliable downstream measurement in the current workflow, whereas length, shift, tortuosity, and curvature should be interpreted more cautiously.

**Figure 7:**
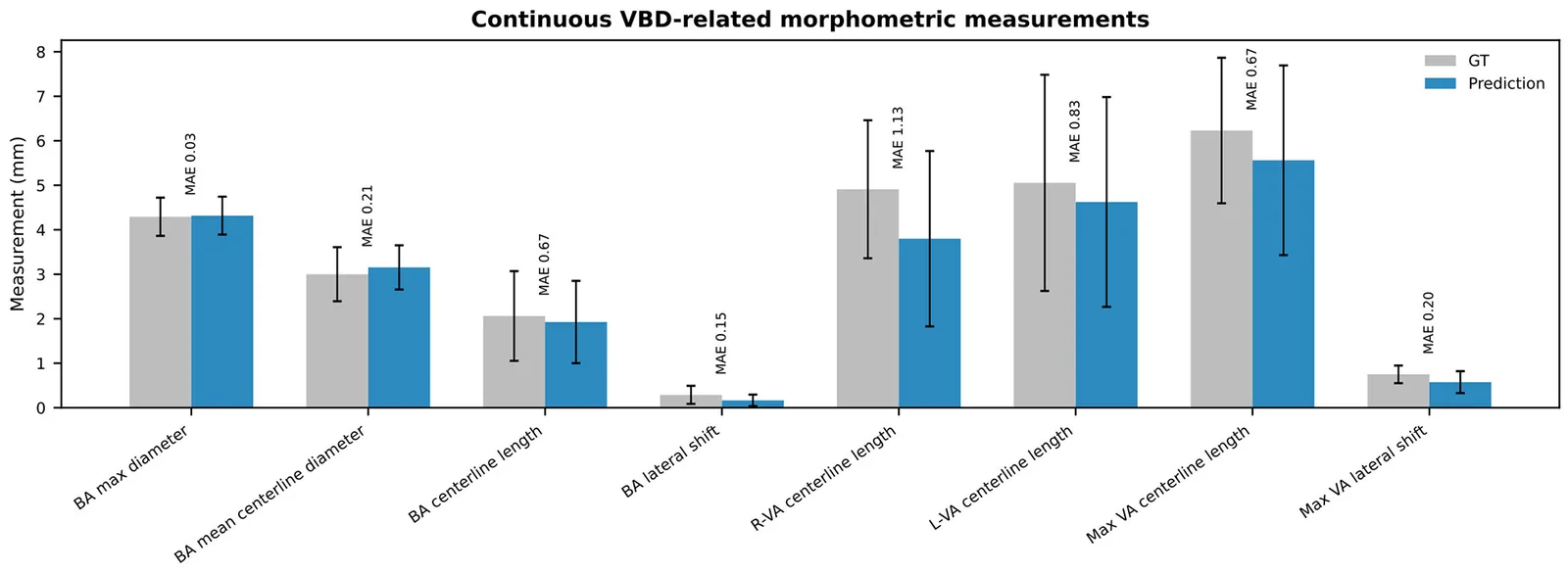
Continuous VBD-oriented morphometric measurements derived from ground-truth and prediction masks. The figure compares GT-derived and prediction-derived values for BA diameter, BA centerline measurements, VA centerline length, and VA lateral shift. Diameter-related measurements show close agreement, supporting the use of the proposed workflow for diameter-based VBD-oriented assessment. Length and shift are computed from annotated vessel segments and should be interpreted as segmentation-derived geometric measurements rather than complete clinical anatomical measurements of the entire vertebrobasilar course.

Continuous VBD-oriented measurements are summarized in Table 5, and aggregate measurement errors are reported in Table 6.

**Table 5:** Continuous VBD–oriented morphometric measurements.

Measurement	GT mean ±std	Pred mean ±std	MAE mean ±std	Interpretation
BA max diameter (mm)	4.2921 ± 0.4300	4.3173 ± 0.4261	0.0252 ± 0.0667	Diameter–based VBD–oriented index
BA mean centerline diameter (mm)	3.0005 ± 0.6075	3.1538 ± 0.4966	0.2120 ± 0.1067	Diameter–related vessel caliber index
BA centerline length (mm)	2.0617 ± 1.0083	1.9261 ± 0.9255	0.6707 ± 0.4780	Segment–level geometric descriptor
BA lateral shift (mm)	0.2897 ± 0.2032	0.1656 ± 0.1296	0.1516 ± 0.1851	Segment–level geometric descriptor
R–VA centerline length (mm)	4.9102 ± 1.5505	3.7987 ± 1.9715	1.1320 ± 1.5534	Segment–level geometric descriptor
L–VA centerline length (mm)	5.0536 ± 2.4292	4.6249 ± 2.3571	0.8257 ± 0.8280	Segment–level geometric descriptor
Max VA centerline length (mm)	6.2307 ± 1.6349	5.5617 ± 2.1313	0.6690 ± 1.0297	Segment–level geometric descriptor
Max VA lateral shift (mm)	0.7502 ± 0.1981	0.5754 ± 0.2461	0.2014 ± 0.1948	Segment–level geometric descriptor

GT denotes measurements derived from ground–truth masks, and Pred denotes measurements derived from two–stage VB–ROI predictions. Length and shift values are computed from the available annotated vessel segments and should not be interpreted as complete clinical vertebrobasilar measurements.

**Table 6:** Aggregate morphometric measurement error of the two–stage VB–ROI workflow.

Metric family	MAE	Pearson r	ICC
Max diameter	0.0464 mm	0.9924	0.9919
Mean centerline diameter	0.1553 mm	0.9574	0.9413
Centerline length	0.8761 mm	0.8363	0.8144
Lateral shift	0.2213 mm	0.4368	0.3803
Tortuosity	0.1072	0.1593	0.1564
Mean curvature	0.5011	0.2928	0.2952
Max curvature	0.2184	0.4999	0.4948

The values summarize available vessel–level measurements within each metric family.

### 4.6 Threshold-based consistency analysis

Table 7 summarizes the main threshold-based consistency results. To avoid overstating clinical validation, we report these results as consistency between prediction-derived and reference-derived morphometric flags within the current validation split. For BA diameter greater than 4.5 mm and overall VBD-oriented positivity, prediction-derived morphometry reproduced the reference-derived flags exactly within this validation split. This indicates internal consistency of the diameter-based morphometric endpoint under the present data and annotation conditions, rather than established clinical accuracy.

**Table 7:** Main threshold–based consistency results of VBD–oriented morphometric analysis.

Threshold	Sens.	Spec.	Acc.	Kappa
BA diameter > 4.5 mm	1.0000	1.0000	1.0000	1.0000
Any VBD–oriented positive flag	1.0000	1.0000	1.0000	1.0000

These results should be interpreted as preliminary agreement analysis rather than definitive clinical validation.

The complete threshold results are shown in Table 8. Several length- and displacement-related endpoints had no positive reference cases in the current validation split. Their high specificity therefore mainly reflects false-positive control under the absence of positive references, and does not constitute validation of those criteria. Establishing the clinical validity of length-, displacement-, and composite VBD criteria would require larger, VBD-enriched, multi-center cohorts with complete vertebrobasilar annotations; this lies beyond the scope of the present feasibility study and is deferred to future work.

**Table 8:** Complete threshold–based consistency results of VBD–oriented morphometric analysis.

Threshold	Sens.	Spec.	Acc.	Kappa
BA diameter > 4.5 mm	1.0000	1.0000	1.0000	1.0000
BA length > 29.5 mm	N/A	1.0000	1.0000	N/A
BA shift > 10 mm	N/A	1.0000	1.0000	N/A
VA length > 23.5 mm	N/A	1.0000	1.0000	N/A
VA shift > 10 mm	N/A	1.0000	1.0000	N/A
BA diameter ≥ 5.3 mm	N/A	1.0000	1.0000	N/A
Any VBD–oriented positive flag	1.0000	1.0000	1.0000	1.0000

N/A indicates that sensitivity or kappa was unavailable because no positive reference case was present for the corresponding endpoint. These N/A entries do not represent missing data; instead, they indicate that sensitivity or kappa calculation was not applicable and reflect appropriate false-positive control in the corresponding subgroup analysis.

### 4.7 Failure case analysis

Figure 8 analyzes residual failure patterns of the two-stage VB-ROI workflow. Although ROI focusing substantially improves segmentation stability, remaining errors occur mainly at distal vessel endpoints, weak-signal VA segments, and regions where thin vessels are adjacent to small side branches. These errors may have limited effect on diameter estimation but can influence centerline-dependent measurements such as length, lateral shift, tortuosity, and curvature. This observation further supports our interpretation that diameter-related VBD-oriented measurements are more robust than higher-order geometric indices in the current setting.

**Figure 8:**
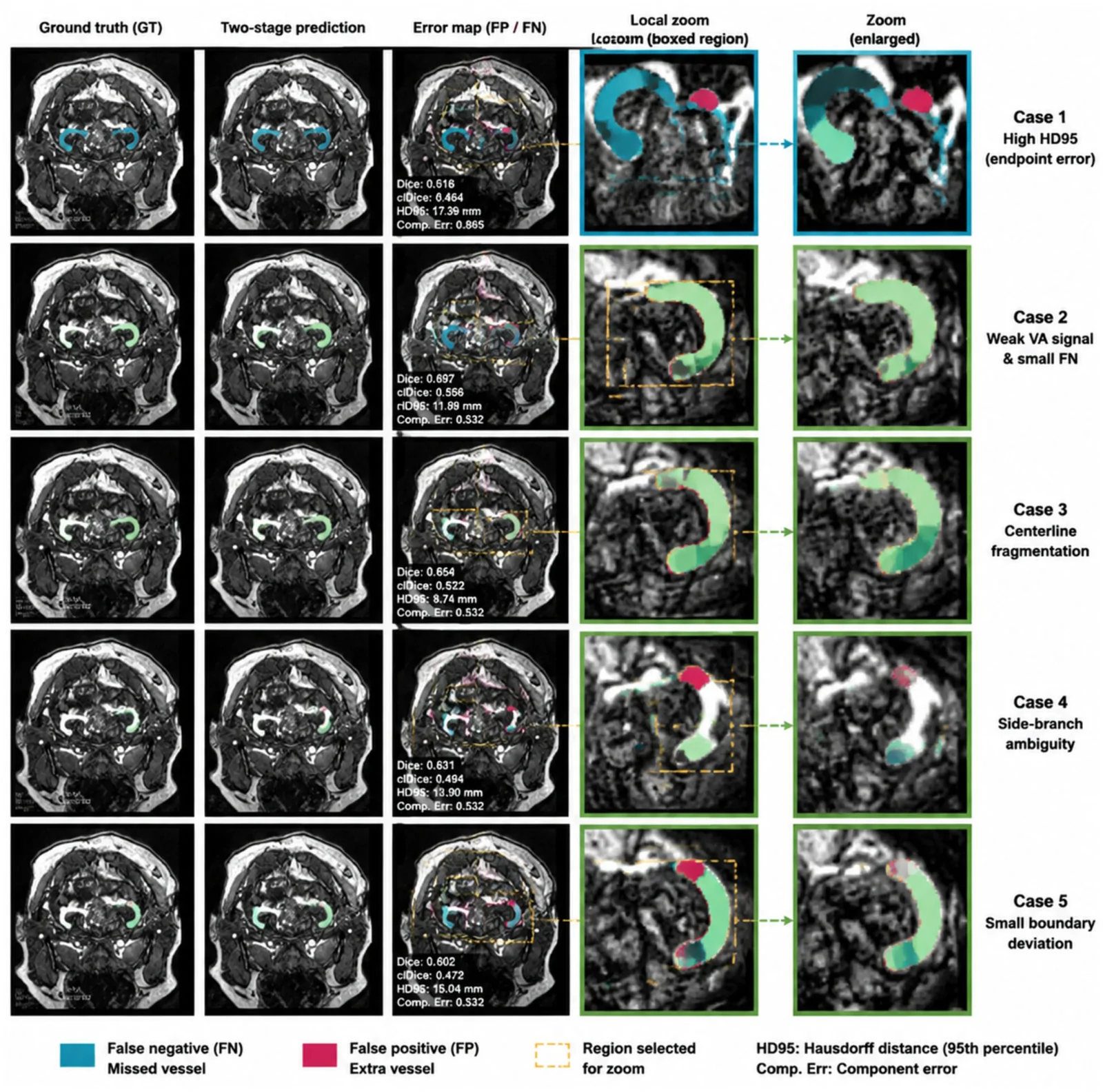
Failure case analysis of the two-stage VB-ROI workflow. Representative cases are selected according to low Dice, low clDice, high HD95, or high component error. Each row shows the ground truth, two-stage prediction, error map, and local zoom. False negatives are shown in cyan and false positives in magenta. Enlarged views highlight the regions indicated by dashed boxes. Residual errors are mainly associated with distal vessel endpoints, weak VA signal, local centerline fragmentation, side-branch ambiguity, or small boundary deviations that disproportionately affect HD95.

In subgroup analyses where no positive reference cases were available for a specific endpoint, sensitivity and kappa values were marked as N/A because these metrics were not applicable. These entries should be interpreted as reflecting appropriate false-positive control rather than missing measurements.

## 5 Discussion

The main finding of this study is that restricting fine vessel segmentation to a vertebrobasilar ROI substantially improves the delineation of the BA and bilateral VAs compared with direct full-image segmentation. When the basilar artery (BA) and bilateral vertebral arteries (VAs) are segmented within the full magnetic resonance angiography (MRA) space, the model must concurrently address target localization, vessel discrimination, and precise boundary delineation within a vast field that is predominantly characterized by irrelevant background elements, adjacent non-target vasculature, and weak-signal vessel segments. This scenario is poorly aligned with VBD-oriented morphometric analysis, where the clinically relevant structures consist of a limited number of thin, elongated, and spatially localized vessels. Minor discontinuities, endpoint errors, or boundary outliers that may seem insignificant in voxel-overlap evaluations can substantially impact centerline extraction, local caliber estimation, lateral displacement, tortuosity, and curvature. Consequently, the proposed workflow should not be viewed merely as an attempt to improve segmentation scores; rather, it provides a more appropriate spatial and anatomical setting for vertebrobasilar vessel segmentation and downstream morphometric quantification.

A potential concern is that the proposed workflow may be perceived as a cascade or stacking of two segmentation models. However, this is not the intended methodological role of the design. The two stages address distinct and non-overlapping tasks. The first-stage full-image nnU-Net provides a coarse anatomical localization prior and generates a prediction-derived VB-ROI. Its output is not utilized as the final vessel mask, does not fuse with the second-stage output, and is not employed for morphometric measurement. Conversely, the second-stage nnU-Net conducts fine BA/R-VA/L-VA segmentation within the anatomically focused field of view, with only the restored second-stage prediction being used for segmentation evaluation and morphometric analysis. Therefore, the workflow is more accurately described as a decomposition of tasks into localization, segmentation, and morphometry, rather than model stacking. By minimizing irrelevant anatomical context and enhancing the relative spatial prominence of the target vertebrobasilar vessels, the VB-ROI setting transforms an ill-conditioned full-image segmentation problem into a well-conditioned fine delineation problem.

The comparison among the full-image nnU-Net, ground truth (GT)-based VB-ROI, and prediction-derived VB-ROI settings further substantiates this interpretation. The GT-based VB-ROI setting functions as an oracle for spatial-context control rather than as a deployable method. Its primary purpose is to isolate the benefits of concentrating the segmentation field of view on the vertebrobasilar region. Conversely, the prediction-derived VB-ROI setting exemplifies the practical workflow devoid of test-time ground truth. The comparable performance observed between these two ROI settings suggests that the significant performance improvement is primarily due to spatial-context focusing rather than merely the incorporation of an additional network. In essence, the initial stage does not necessitate voxel-perfect segmentation; it only needs to localize a sufficiently inclusive vertebrobasilar field of view. Once the target basilar artery (BA), right vertebral artery (R-VA), and left vertebral artery (L-VA) anatomies are preserved within the ROI, the second-stage model can recover more stable vessel boundaries and centerline structures. This interpretation is further corroborated by the ROI margin sensitivity analysis, which indicates that complete target-vessel coverage can be achieved with a focused crop volume following physical-margin expansion.

From a morphometric perspective, the proposed workflow demonstrates the highest reliability for diameter-related measurements. This finding is significant because the basilar artery (BA) diameter is one of the most frequently utilized imaging criteria oriented toward vessel diameter (VBD). The estimation of diameter primarily hinges on the accuracy of local boundaries. In contrast, measurements such as length, lateral shift, tortuosity, and curvature are more sensitive to factors including endpoint definition, centerline extraction, vessel continuity, and the anatomical scope of the available annotations. Consequently, these indices impose stricter requirements than mere voxel-level overlap or local boundary accuracy. In the context of the present dataset, they should be interpreted as geometric descriptors derived from segmentation of annotated vessel segments rather than as comprehensive clinical measurements of the entire vertebrobasilar course. This distinction is crucial to prevent over-interpretation. Under the current data and annotation conditions, the workflow facilitates technically reliable diameter-oriented morphometric quantification. However, a thorough characterization of dolichosis-related elongation, displacement, tortuosity, and curvature necessitates more comprehensive annotations and larger cohorts enriched with VBD.

The lower agreement observed for tortuosity and curvature compared with diameter-related measurements is likely related to the inherent sensitivity of higher-order geometric descriptors to centerline extraction and endpoint definition. Unlike diameter estimation, which mainly depends on local vessel boundary accuracy, tortuosity and curvature rely on the global trajectory and local directional changes of the extracted centerline. Small variations in segmentation boundaries, vessel continuity, or endpoint localization may therefore be amplified during skeletonization-based calculations. Accordingly, these higher-order morphometric indices should be interpreted cautiously in the current study and should not be considered as definitive clinical measurements without further validation using more comprehensive annotations and larger cohorts.

The threshold-based consistency analysis should be interpreted with caution. Although a BA diameter greater than 4.5 mm and overall VBD-oriented positivity achieved complete agreement in the current validation split, several length- and displacement-related thresholds lacked positive reference cases. Consequently, these results should be considered as a preliminary internal consistency analysis rather than a clinical validation of diagnostic criteria. The high specificity observed in endpoints without positive reference cases primarily reflects false-positive control within the current validation distribution and does not establish sensitivity across the entire VBD spectrum. Future studies should focus on evaluating calibration, diagnostic thresholds, and clinical utility in larger, multi-center cohorts enriched with VBD cases, complete vertebrobasilar annotations, and independent clinical outcome labels.

Although the proposed framework demonstrated strong performance within the current dataset, this study was evaluated using a fixed split of the publicly available TopBrain-MRA dataset, and independent external validation was not conducted. Vascular segmentation models may experience performance degradation when applied to data acquired from different institutions, scanner manufacturers, magnetic field strengths, or acquisition protocols. Therefore, multi-center external validation using independent cohorts with heterogeneous imaging conditions is required to further assess the generalizability and clinical applicability of the proposed workflow.

This study presents several limitations. Firstly, the evaluation was conducted on a limited validation split; therefore, larger multi-center datasets are necessary to assess generalization across different scanners, acquisition protocols, imaging quality, and patient populations. Secondly, the available annotations were primarily focused on BA/R-VA/L-VA target vessel segments, which limits the interpretation of global length, displacement, tortuosity, and curvature measurements that require anatomically complete and consistently defined vessel endpoints. Thirdly, clinical outcome labels were not available, indicating that the present work should be regarded as VBD-oriented imaging quantification rather than a prediction of posterior-circulation-infarction risk. Fourthly, while the comparison between GT-based and prediction-derived VB-ROI settings supports the significance of spatial-context focusing, additional controls such as fixed-location ROI cropping, atlas-based ROI selection, or alternative localization strategies would further elucidate the contribution of subject-specific localization. Finally, external validation on prospectively recruited cohorts with outcome labels is essential before the workflow can be interpreted as a clinically validated diagnostic or prognostic tool.

Overall, this study suggests that the VBD-oriented MRA analysis is enhanced by redefining the segmentation task to align with the anatomical and quantitative requirements of vertebrobasilar morphometry. The proposed workflow focuses on VB-ROI, differentiating between coarse anatomical localization and fine vessel delineation, thereby linking the restored segmentation mask to subsequent morphometric assessments. This design does not aim to introduce a novel segmentation architecture or a clinically validated diagnostic system. Instead, it provides a task-specific technical framework for generating morphometry-ready BA/R-VA/L-VA masks, currently offering the strongest support for diameter-related measurements and outlining a clear pathway for the future validation of centerline-dependent and outcome-related VBD metrics.

Importantly, the present study should be interpreted as a technical validation of an automated segmentation-to-morphometry framework rather than a prognostic study evaluating clinical outcomes. Although VBD has been associated with posterior circulation ischemic events and brainstem compression in previous clinical studies, the current investigation does not include outcome prediction or longitudinal clinical follow-up. The primary objective was to assess whether the proposed workflow can generate reliable vertebrobasilar vessel segmentations and reproducible morphometric measurements from MRA data, thereby providing a technical foundation for future clinical studies.

## 6 Conclusion

This study presents a segmentation-to-morphometry workflow focused on VB-ROI for VBD-oriented MRA analysis. Rather than introducing a new network architecture, the proposed workflow reformulates BA/R-VA/L-VA delineation as a decomposition of localization and segmentation tasks. In this approach, a full-image nnU-Net is utilized to generate a coarse vertebrobasilar localization prior, while a ROI nnU-Net is employed for fine vessel segmentation within an anatomically focused field of view. Compared to direct full-image nnU-Net, this ROI-focused workflow significantly improves the segmentation of BA, R-VA, and L-VA, with particularly notable improvements observed for the vertebral arteries. The comparable performance between prediction-derived and ground truth (GT)-based VB-ROI settings further suggests that robust ROI focusing can be achieved without relying on test-time ground truth. The restored segmentation masks demonstrated strong agreement for diameter-related morphometric measurements and supported preliminary threshold-based consistency analysis. However, centerline-dependent indices require further validation with more comprehensive annotations and VBD-enriched cohorts. Overall, the VB-ROI-focused segmentation offers a technically feasible strategy for generating morphometry-ready vertebrobasilar masks from MRA, particularly for diameter-oriented quantification, and provides a task-specific methodological basis for future clinical validation related to VBD and posterior circulation infarction.

## Data Availability

The datasets analyzed in this study are publicly available from the TopBrain Challenge Data Release on Zenodo (doi: 10.5281/zenodo.16878417), subject to the applicable data-use terms. No new datasets were generated in this study.
